# N-Doped Graphene Quantum Dots Confined within Silica Nanochannels for Enhanced Electrochemical Detection of Doxorubicin

**DOI:** 10.3390/molecules28186443

**Published:** 2023-09-05

**Authors:** Chaoyan Zhang, Xiaoyu Zhou, Fei Yan, Jing Lin

**Affiliations:** 1 Key Laboratory of Surface & Interface Science of Polymer Materials of Zhejiang Province, Department of Chemistry, Zhejiang Sci-Tech University, Hangzhou 310018, China; 202130107346@mails.zstu.edu.cn (C.Z.); 202120104191@mails.zstu.edu.cn (X.Z.); 2 The First Affiliated Hospital of Guangxi University of Chinese Medicine, Nanning 530200, China

**Keywords:** N-doped graphene quantum dots, vertically ordered mesoporous silica films, doxorubicin, electrochemical sensors

## Abstract

Herein, we describe a fast and highly sensitive electrochemical sensor for doxorubicin (DOX) detection based on the indium tin oxide (ITO) modified with a binary material consisting of vertically-ordered mesoporous silica films (VMSFs) and N-doped graphene quantum dots (NGQDs). VMSFs, with high permeability and efficient molecular transport capacity, is attached to the ITO electrode via a rapid and controllable electrochemical method, which can serve as a solid template for the confinement of numerous NGQDs through facile electrophoresis. By virtue of the excellent charge transfer capacity, π-π and electrostatic preconcentration effects of NGQDs, as well as the electrostatic enrichment ability of VMSF, the presented NGQDs@VMSF/ITO shows amplified electrochemical signal towards DOX with a positive charge, resulting in good analytical performance in terms of a wide linear range (5 nM~0.1 μM and 0.1~1 μM), high sensitivity (30.4 μA μM^−1^), and a low limit of detection (0.5 nM). Moreover, due to the molecular sieving property of VMSF, the developed NGQDs@VMSF/ITO sensor has good selectivity and works well in human serum and urine samples, with recoveries of 97.0~109%, thus providing a simple and reliable method for the direct electrochemical analysis of DOX without complex sample pretreatment procedures.

## 1. Introduction

Doxorubicin (DOX) is a widely used anthracycline anticancer drug that is effective in treating breast, lung, liver, and ovarian cancer [1,2,3]. Its mechanism of action involves inhibiting DNA replication and transcription in cancer cells [4,5]. However, DOX has several adverse effects on the human body, including nausea, vomiting, liver failure, local tissue necrosis, and potential heart failure [6,7]. Therefore, it is crucial to closely monitor the dosage of DOX in human biological fluids and further regulate its concentration. Various methods have been developed to quantify DOX in biological samples, such as high-performance liquid chromatography [8], fluorometry [9,10], electrophoresis [11], and electrochemical sensors [12]. Among these methods, electrochemical techniques offer a simple, sensitive, and cost-effective approach without the need for a professional operator or large-scale instrument, which has been widely used for trace analysis of DOX in biological samples [12,13]. DOX bearing hydroquinone and quinone moieties can give rise to the redox signals at the electrode surface, resulting in the design of various electrochemical detection strategies using many different nanomaterials [14,15]. However, the presence of biomolecules in biological fluids and other electrochemically active compounds often interferes with the effective electron transfer between DOX and electrode, compromising the sensitivity, repeatability, and reliability of electroanalytical sensors. 

Nanostructured materials with their unique features (high surface area and nanoconfinement effects) have received a great deal of interest compared to the bulk counterparts. [16,17,18,19]. To date, vertically-ordered mesoporous silica films (VMSFs) have gained increasing attention due to their unique permselectivity and anti-fouling abilities in complex samples [20,21,22,23]. Benefiting from perpendicularly ordered channel structure, the ultrasmall and adjustable pore size (2~11 nm), as well as the high density of silanol groups on the walls, VMSFs can serve as a preconcentrated material on the electrode surface for analytes of interest through various interactions (e.g., electrostatic, size exclusion, and lipophilicity) between analytes or probe and silica nanochannels, effectively increasing the analytical performance [24,25,26]. Owing to the uniform pore size, as well as large specific surface area and good mechanical stability, VMSFs have emerged as superior protective layer on the electrode surface and simultaneously allows the effective diffusion of small molecules (analytes or probes), greatly minimizing contamination of the complex matrix on the electrode and maintaining the electrode performance [27,28,29,30,31]. Moreover, VMSFs with many tiny nanochannels can confine various nanomaterials, such as metals [32,33,34], polymers [35], graphene quantum dots (GQDs) [36], and electrochemical probes [37,38,39], to fabricate a functional sensing interface. These exceptional characteristics of VMSFs provide a universal anti-fouling electroanalytical platform for direct determination in complicated real matrix [40,41]. 

GQDs are a kind of zero-dimensional materials and composed of small graphene fragments, and have been widely employed for various fluorescent or colorimetric sensors due to their unique characteristics in terms of large specific surface area, good water solubility, excellent electron mobility, and high biocompatibility [42,43,44,45,46]. Since metal ions can induce the aggregation of GQDs through the coordination interaction between metal ions and functional groups of GQDs, a great deal of fluorescent sensors based on GQDs have been reported for the detection of Hg^2+^, Cu^2+^, and Fe^3+^ using fluorescence quenching responses [47,48,49]. In addition, GQDs have been utilized to modify the electrode surface for enhanced electrochemical detection. As our group reported previously, GQDs can be confined in an ultrasmall space of perpendicularly aligned silica nanochannels through electrophoresis and display ultrasensitive and rapid detection for metal ions and dopamine [36].

**Scheme 1:**
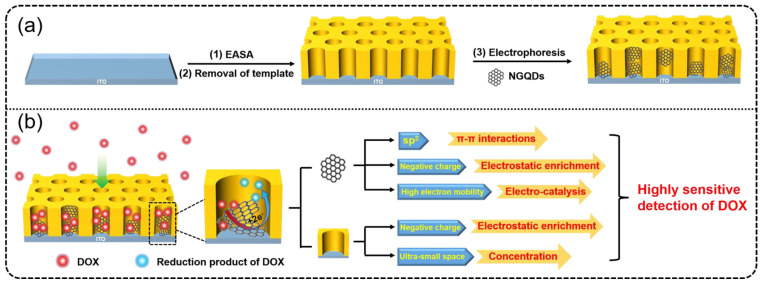
Schematic diagram of the preparation process of the NGQDs@VMSF/ITO electrode (**a**) and highly sensitive sensing mechanism of NGQDs@VMSF/ITO electrode for DOX (**b**).

In this work, we report a simple and highly sensitive electrochemical method for DOX detection in human serum and urine samples based on the indium tin oxide (ITO) electrode modified with VMSF confining N-doped graphene quantum dots (NGQDs) into the silica nanochannels. VMSFs, possessing high permeability and efficient molecular transport capacity, can act as a solid template for the physical confinement of NGQDs via facile electrophoresis. The achieved NGQDs@VMSF/ITO sensor combines the advantages of VMSF and NGQDs (Scheme 1): (1) electrostatic preconcentration effects of NGQDs and VMSF, as well as the π-π interaction of NGQDs for the enrichment of DOX with positive charge to the electrode surface; (2) excellent charge transfer capacity of NGQDs for facilitating the electron transfer between DOX and the underlying electrode; (3) tiny spaces in nanochannels of VMSF for amplifying the electrochemical signals of DOX. The constructed NGQDs@VMSF/ITO sensor shows good analytical performance for DOX determination. Furthermore, thanks to the molecular sieving property of VMSF and cost-effective electrode fabrication, the designed NGQDs@VMSF/ITO sensor enables the direct analysis of DOX in a complex matrix (human serum and urine), making it a promising candidate for reliably monitoring DOX content in clinical diagnosis.

## 2. Results and Discussion

### 2.1. Characterization of NGQDs

**Figure 1:**
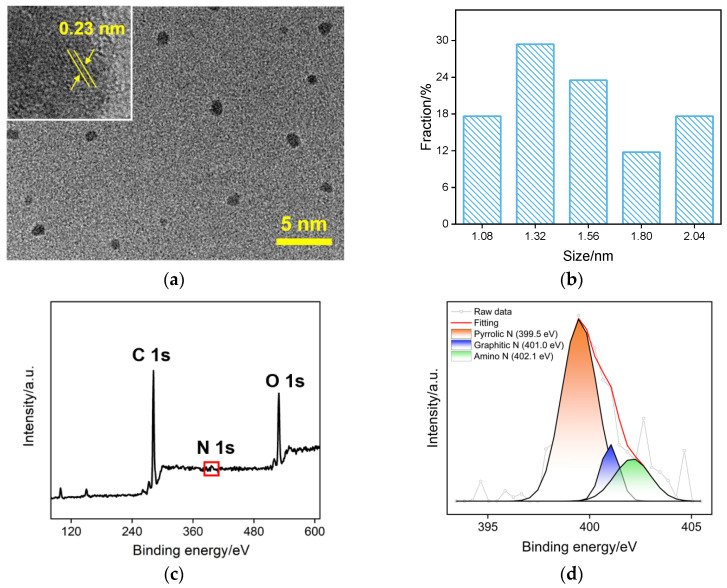
(**a**) TEM image of the as-prepared NGQDs. Inset is high-resolution TEM image with indicated lattice parameter and (**b**) size distribution (bottom) of NGQDs. (**c**) XPS survey spectrum of NGQDs. (**d**) High-resolution N 1s of the synthesized NGQDs.

NGQDs used in this work were synthesized by a one-step hydrothermal method, which was firstly studied using transmission electron microscopy (TEM) and X-ray photoelectron spectroscopy (XPS). As shown in Figure 1a,b, NGQDs have a uniform size of approximately 1.5 nm on average. Their lattice spacing is 0.23 nm, which is assigned to the graphene (100) plane (inset of Figure 1a). The survey XPS and high-resolution N 1s spectra of NGQD show three distinct peaks of C 1s, N 1s, and O 1s (Figure 1c,d), confirming the presence of amino and hydroxyl groups.

### 2.2. Characterization of VMSF/ITO before and after Physical Confinement of NGQDs

**Figure 2:**
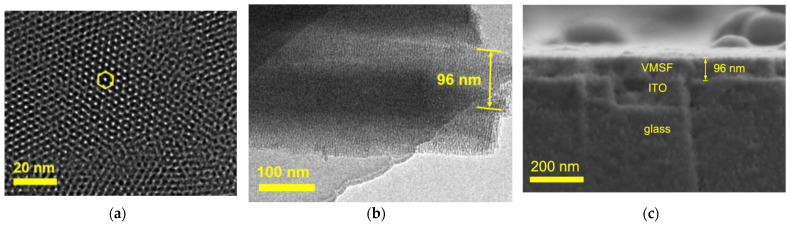
Electron microscopy measurements of VMSF/ITO electrode: (**a**) top-view TEM, (**b**) cross-sectional view TEM, and (**c**) cross-sectional view SEM.

**Figure 3:**
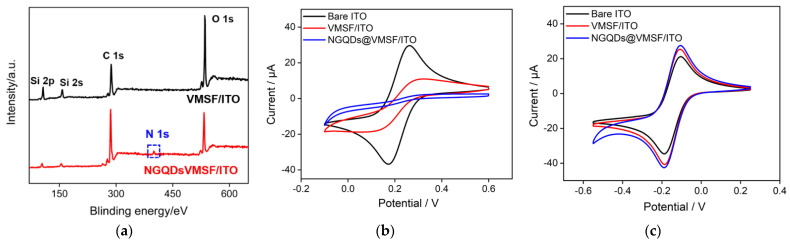
(**a**) XPS survey spectrum of NGQDs. CV responses of 0.5 mM K_3_[Fe(CN)_6_] (**b**) and [Ru(NH_3_)_6_]Cl_3_ (**c**) at the bare ITO (black curve), VMSF/ITO (red curve), and NGQDs@VMSF/ITO (blue curve) electrodes in 50 mM KHP solution.

VMSF grown on the ITO surface was characterized by TEM, showing a uniform pore size of 2~3 nm and perpendicularly ordered structure with a thickness of 96 nm (Figure 2a,b). The cross-section of VMSF contains three layers from top to bottom, namely VMSF, ITO, and glass, which is also confirmed by scanning electron microscopy (SEM) (Figure 2c). The size of NGQDs (~2.0 nm) is similar to the pore size of the silica nanochannels, allowing the confinement of NGQDs into the nanochannels of VMSF to form NGQDs@VMSF/ITO. XPS and energy dispersive spectrometer (EDS) analyses further prove the confinement of NGQDs in the nanochannels through the detection of the characteristic peak of nitrogen element (Figure 3a and Figure S1). In addition, electrochemical technique was used to investigate the as-prepared NGQDs@VMSF/ITO electrode. In the cyclic voltammetry (CV) curve of bare ITO, displayed in Figure 3b,c, well-defined reversible redox peaks are observed, which is assigned to the electrochemical reaction of K_3_[Fe(CN)_6_] and Ru(NH_3_)_6_]Cl_3_ probes, respectively. Interestingly, the VMSF/ITO electrode exhibits enhanced electrochemical signal for cationic Ru(NH_3_)_6_]Cl_3_ but decreased the signal for anionic K_3_[Fe(CN)_6_] compared with bare ITO. This case indicates that VMSF is a good electrode-modified material for the detection of positively charged analytes because of the negative charges of silanol groups on the silica walls and apparent electrostatic effect in the ultrasmall pore size of VMSF in the measured experimental conditions. After the confinement of NGQDs into the nanochannels, NGQDs@VMSF/ITO exhibits further remarkable electrostatic permselectivity, which is attributed to the negative charge of NGQDs in the experimental conditions [29], and also indicates the successful preparation of the NGQDs@VMSF/ITO sensor.

### 2.3. Enhanced Electrochemical Response of DOX on NGQDs@VMSF/ITO

**Figure 4:**
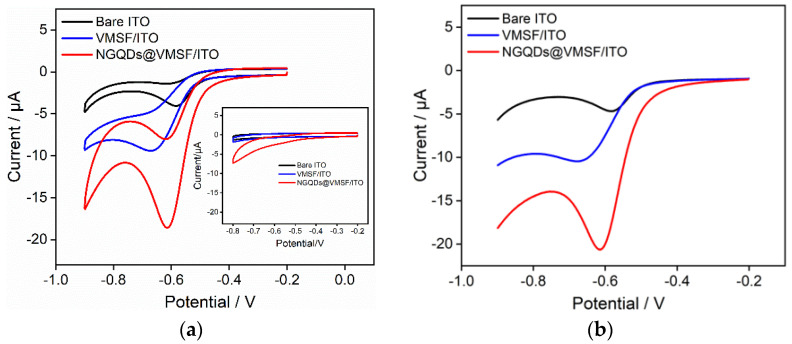
CV (**a**) and LSV (**b**) plots of 1 μM DOX at the bare ITO, VMSF/ITO, and NGQDs@VMSF/ITO electrodes in 0.01 M PBS (pH = 7.0) solution. Inset in (**a**) is these three electrodes in 0.01 M PBS (pH = 7.0) solution in the absence of DOX. The scan rate for CV tests was 50 mV s^–1^.

The electrochemical behavior of DOX during potential scan from −0.2 to −0.9 V at the bare ITO, VMSF/ITO, and NGQDs@VMSF/ITO electrodes was investigated, respectively, and the results were recorded in Figure 4. These three types of electrode exhibit no Faradic signals in blank PBS (inset of Figure 4a), but show an apparent irreversible cathodic peak for DOX (Figure 4a), indicating the occurrence of the electrochemical reduction process of DOX at the electrode surface. The cathodic peak current of DOX at the VMSF/ITO electrode is significantly higher than that at the bare ITO electrode, which is thanks to the electrostatic enrichment of DOX (p*K*_a_ = 8.22) by the negatively charged properties of VMSF. Although the insulating property, VMSF remains highly permeable for the accessible diffusion of DOX due to the high porosity and ultrathin features. In addition, NGQDs@VMSF/ITO gives more sensitive cathodic peak current for DOX and the obtained current value is approximately three times higher than that obtained at the VMSF/ITO electrode (Figure 4b), which is due to the proconcentration effect in terms of electrostatic attraction and π-π interaction between NGQDs and DOX, as well as the enhanced conductivity and electron mobility of NGQDs. Therefore, the fabricated NGQDs@VMSF/ITO electrode combines the advantages of VMSF and NGQDs, displaying a good electrochemical response for the detection of DOX.

### 2.4. Optimized Conditions for DOX Determination

**Figure 5:**
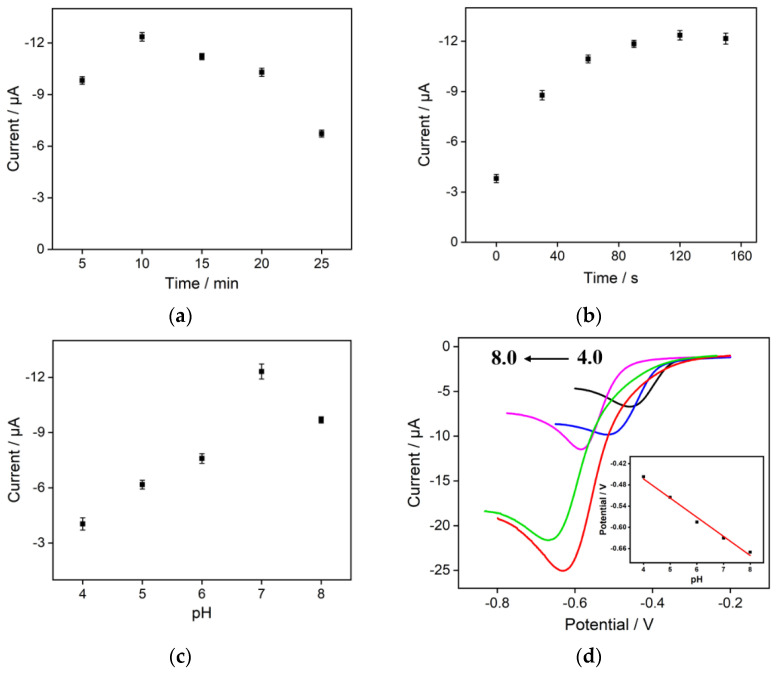
The cathodic current signal of 1 μM DOX at the NGQDs@VMSF/ITO electrode in 0.01 M PBS (pH = 7.0) solution under different electrophoresis time (**a**) and preconcentration time (**b**). (**c**,**d**) Effect of pH values of 0.01 M PBS on the electrochemical signal of 1 μM DOX at the NGQDs@VMSF/ITO electrode, including cathodic peak currents (**c**) and cathodic peak (**d**) in 0.01 M PBS (pH = 7.0) solution. Inset in (**d**) shows the dependence of cathodic peak potential on the pH value.

To achieve excellent performance for DOX analysis, detection conditions, including electrophoresis time for fabrication of NGQDs@VMSF/ITO, enrichment time, and pH of the supporting electrolyte, were optimized. Figure 5a shows the cathodic current responses of 1 μM DOX at the NGQDs@VMSF/ITO electrodes prepared with different electrophoresis times. In the range of 5~10 min, the obtained current value increases with increasing electrophoresis time. When the electrophoresis time is 10 min, the obtained signal value is the largest and the current response gradually decreases after 10 min. This reduced signal under longer electrophoresis time may be due because there are too many NGQDs in the nanochannel to compete with DOX in the space. Therefore, 10 min is selected as the optimal electrophoresis times for the fabrication of NGQDs@VMSF/ITO in this study. As shown in Figure 5b, the cathodic peak current increases with the enrichment time, finally reaching the equilibrium at 120 s. Thus, 120 s is selected as the optimal preconcentration time in the following experiments. The pH of PBS was also optimized; it can be seen from Figure 5c that the magnitude of cathodic peak current increases gradually with the increasing pH value at first and is maximal at pH 7.0, which is due to the pronounced negative charges on the silica wall. However, the obtained current value decreases slightly at pH 8.0, which is attributed to the instability of VMSF in an alkaline environment. Therefore, pH 7 is the optimal condition for the quantification of DOX. In addition, Figure 5d reveals the LSV responses of the NGQDs@VMSF/ITO electrode towards 1 μM DOX at different pH values of 0.01 M PBS in the range from 4.0 to 8.0, yielding a linear relationship between the pH values of PBS and cathodic peak potential (*E*_pc_) (inset of Figure 5d). *E*_pc_ gradually shifts negatively with an increase in pH and displays a good linear relationship with pH (*E*_pc_ = −0.0541pH − 0.247, *R*^2^ = 0.994). The slope (Δ*E*_pc_/ΔpH) of the linear regression equation was −54.1 mV/pH, demonstrating that the number of protons and electrons involved in the electrochemical reaction of DOX at the NGQDs@VMSF/ITO electrode was equal.

### 2.5. Electrochemical Detection of DOX Using NGQDs@VMSF/ITO

**Figure 6:**
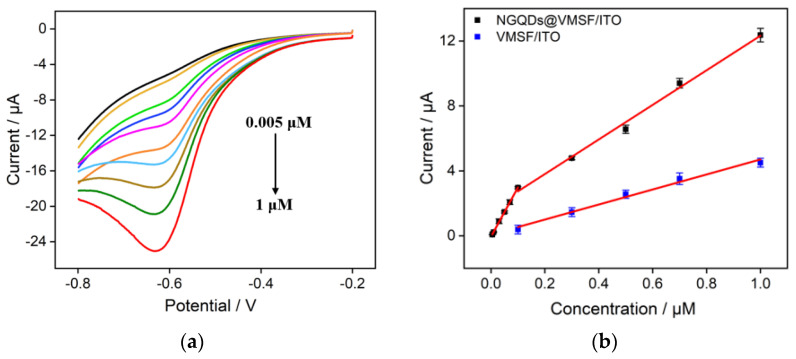
(**a**) LSV response of DOX at the NGQDs@VMSF/ITO electrode in 0.01 M PBS solution (pH 7.0). The concentrations of DOX are 0.005 μM, 0.01 μM, 0.03 μM, 0.05 μM, 0.07 μM, 0.1 μM, 0.3 μM, 0.5 μM, 0.7 μM, and 1 μM, respectively. (**b**) The linear relationship between the cathode peak currents and DOX concentration obtained at both NGQDs@VMSF/ITO and VMSF/ITO electrodes. The concentrations of DOX at the VMSF/ITO electrode are 0.1 μM, 0.3 μM, 0.5 μM, 0.7 μM, and 1 μM, respectively.

Figure 6a illustrates the LSV responses of DOX with different concentrations at the NGQDs@VMSF/ITO electrode in 0.01 M PBS. The results show that the cathodic peak current gradually increases with the addition of DOX in the range from 5 nM to 0.1 μM. As presented in Figure 6b, the as-prepared NGQDs@VMSF/ITO sensor demonstrates a good linear relationship between the cathodic peak current (*I*, μA) and DOX concentration (*C*_DOX_, μM), with two linear ranges, namely 5 nM to 0.1 μM and 0.1 to 1 μM. The resulting linear fitting equations in the low and high concentration ranges are *I* (μA) = 30.4 *C*_DOX_ (μM) −0.0530 (*R*^2^ = 0.995) and *I* (μA) = 10.7 *C*_DOX_ (μM) −1.68 (*R*^2^ = 0.993), respectively. The calculated limit of detection (LOD) was 0.5 nM (S/N = 3). The analytical capacity of VMSF/ITO for DOX detection was also evaluated under the same experimental conditions and the results are compared in Figure 6b. Our NGQDs@VMSF/ITO sensor has several apparent advantages over VMSF/ITO in terms of linear range (5 nM~1 μM vs. 0.1~1 μM), sensitivity (30.4 μA μM^−1^ vs. 4.63 μA μM^−1^), and much lower LOD. In addition, Table 1 provides a comparison of the analytical performance of NGQDs@VMSF/ITO for DOX detection with other electrochemical sensors, showing that the developed NGQDs@VMSF/ITO sensor has higher sensitivity, wider linear range, and lower LOD. The fabrication of the NGQDs@VMSF/ITO sensor is cheap and simple, without the need of many materials, along with the disposable property, making the sensor more suitable for a range of practical application.

### 2.6. Selectivity and Anti-Fouling Ability of NGQDs@VMSF/ITO and Real Sample Analysis 

**Figure 7:**
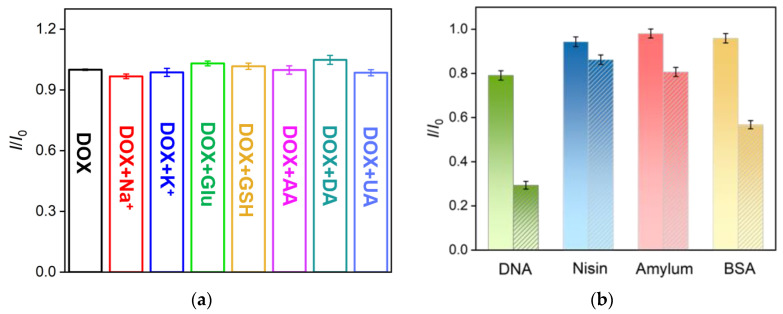
(**a**) The obtained current ratio (*I*/*I*_0_) at the NGQDs@VMSF/ITO electrode for the determination of 1 μM DOX without (*I*_0_) and with (*I*) the added interfering species (Na^+^, K^+^, Glu, GSH, AA, DA, and UA). The concentration of interfering species is 5 μM. (**b**) The peak current ratio (*I*/*I*_0_) on the NGQDs@VMSF/ITO (without shadow) and bare ITO (with shadow) in the presence of 1 μM DOX. *I* and *I*_0_ represent the cathodic peak currents obtained in the present and absence of 10 μg/mL of DNA (green), Nisin (blue), amylum (red), or BSA (yellow).

Selectivity of the developed NGQDs@VMSF/ITO sensor for the detection of DOX was then investigated. Interfering species, including sodium ion (Na^+^), potassium ion (K^+^), glutathione (GSH), glucose (Glu), uric acid (UA) dopamine (DA), and ascorbic acid (AA), may co-exist and interfere with DOX detection in practical analysis, which are similarly mixed with DOX, respectively, and detected by the fabricated NGQDs@VMSF/ITO electrode under optimal experimental conditions. Figure 7a records the cathodic peak current variation (*I*/*I*_0_) of 1 μM DOX at the NGQDs@VMSF/ITO electrode with (*I*) or without (*I*_0_) 5 μM interfering species. It can be found that the obtained cathodic peak current of DOX in the presence of five-fold concentration of distractors is almost identical to the signal of DOX alone, demonstrating the good selectivity of NGQDs@VMSF/ITO sensor. Note that the electroactive species (AA, DA, and UA) can enter the nanochannels of VMSF but do not cause a severe impact on the determination of DOX. This is due to that these species have anodic peaks at the ITO electrode and their potentials are far from the signal of DOX in this work. Additionally, four representative simulated fouling substances—DNA (protamine), peptides (Nisin), macromolecular proteins (BSA), and polysaccharides (amylum)—were selected to evaluate the antifouling ability of NGQDs@VMSF/ITO. Figure 7b presents the ratio of the current responses of the bare ITO and NGQDs@VMSF/ITO electrodes in the presence of 1 μM DOX before (*I*_0_) and after (*I*) 5 min of interaction with the simulated fouling agents. It can be clearly observed that the signal at the bare ITO electrode decreases significantly in the presence of different macromolecular substances, indicating that electrode passivation severely reduced the sensitivity of the bare ITO electrode in the complex matrix. However, the current signal at the NGQDs@VMSF/ITO electrode only slightly decreases, showing the protective effect of NGQDs@VMSF against biofouling. Non-electroactive substances in fouling medium can indeed non-specifically adsorb on the most sensitive electrode surface and cause interferences with the detection of analyte. As for our developed NGQDs@VMSF/ITO sensor, similar non-specific adsorption also occurs, but the transport of DOX, which has a small size, to the underlying ITO electrode through silica nanochannels is effective, eventually guaranteeing the sensitivity of electrochemical sensor. All the above results demonstrate that the designed NGQDs@VMSF/ITO has excellent molecular sieving properties and remains an active electrode surface for the electrochemical analysis of DOX in the presence of inorganic ions, electroactive biological small molecules, and biological macromolecules. Such outstanding analytical performance of NGQDs@VMSF/ITO is attributed to the unique features of VMSF. On the one hand, VMSF, with abundant ultrasmall channels and uniform pore size (2~3 nm), can block the transport of biological macromolecules through a size-exclusion effect. On the other hand, VMSF is rich in silanol groups on the pore walls and provides an electrostatic exclusion effect for positively charged interfering substances. 

Excellent anti-fouling and anti-interference effects of VMSF and amplified signal capacity of NGQDs contribute to the potential of NGQDs@VMSF/ITO in the direct analysis of real samples. Considering that real samples are more complex and produce more matrix effects, we selected human serum and urine as the real samples to examine the analytical capacity of as-prepared NGQDs@VMSF/ITO in fouling medium. The received human serum and urine samples were directly diluted samples using 0.01 M PBS (pH = 7.0) and added with several known concentrations of DOX to obtain mimic detection samples, which were further analyzed by our fabricated NGQDs@VMSF/ITO sensor using the standard addition method. By comparing the detected concentration of DOX achieved from the NGQDs@VMSF/ITO and known concentrations of added DOX, recovery can be obtained to evaluate the accuracy of NGQDs@VMSF/ITO in practical application. As listed in Table 2, satisfactory recoveries (97.0~109%) and small relative standard deviation (RSD, <4.3%) values were obtained, suggesting the developed NGQDs@VMSF/ITO sensor is accurate enough and highly feasible for detecting DOX in actual samples.

## 3. Materials and Methods

### 3.1. Chemicals

Tetraethoxysilane (TEOS, Aladdin, Shanghai, China), cetyltrimethylammonium bromide (CTAB, Aladdin, Shannghai, China), sodium nitrate (NaNO_3_, Hangzhou Gaojing Fine Chemical Reagent, Hangzhou, China), hydrochloric acid (HCl, Hangzhou Shuanglin Chemical Reagent, Hangzhou, China), and ethanol (Aladdin, Shanghai, China) were employed to grow VMSF on the ITO-coated glass surface. 1-Aminopyrene (Aladdin, Shanghai, China) and ammonia water (Hangzhou Gaojing Fine Chemical Reagent (China)) were used for synthesis of NGQDs. Potassium ferricyanide (K_3_[Fe(CN)_6_], Aladdin, Shanghai, China), potassium phthalate monobasic (KHP, Aladdin), and hexaammineruthenium (III) chloride (Ru(NH_3_)_6_Cl_3_, Macklin China, Shanghai, China) were used for electrochemical characterization of NGQDs@VMSF/ITO and VMSF/ITO electrodes. Doxorubicin (DOX), sodium phosphate monobasic dihydrate (NaH_2_PO_4_), glucose (Glu), glutathione (GSH), dopamine (DA), ascorbic acid (AA), uric acid (UA), amylum, bovine serum albumin (BSA), and acetone were received from Aladdin (Shanghai, China). Sodium chloride (NaCl) and potassium chloride (KCl) were bought from the Hangzhou Gaojing Fine Chemical Reagent (Hangzhou, China). Sodium phosphate dibasic dodecahydrate (Na_2_HPO_4_), sodium hydroxide (NaOH), nisin, and fish sperm DNA were obtained from Macklin (Shanghai, China).

### 3.2. Quantitative Detection of DOX Using the Developed NGQDs@VMSF/ITO Electrode

A series of DOX with known concentrations was added to the 0.01 M PBS solution (pH 7.0) and then the NGQDs@VMSF/ITO electrode was used to record the electrochemical signal of DOX under optimal experimental conditions. The enrichment procedure was performed under stirring for 120 s and LSV method was employed to record the electrochemical signals of DOX at room temperature. The cathodic peak currents displayed at −0.6 V were used for the determination of DOX.

The other experimental details about the used materials and instruments, and fabrication procedures of VMSF/ITO and NGQDs@VMSF/ITO electrodes are seen in Supplementary Materials.

## 4. Conclusions

In summary, we have developed a fast and facile electrochemical sensor for the quantitative detection of DOX in human serum and urine samples based on the NGQDs@VMSF/ITO sensor. VMSF bears ultrasmall nanochannels, rendering a solid template for the physical confinement of NGQDs through electrophoresis. Owing to the excellent charge transfer capacity, π-π, and electrostatic preconcentration effects of NGQDs, as well as the electrostatic enrichment and anti-fouling abilities of VMSF, our proposed NGQDs@VMSF/ITO sensor can not only display superior analytical performance for DOX detection with a high sensitivity (30.4 μA μM^−1^) and a rather low limit of detection (0.5 nM), but also demonstrates great potential for pretreatment-free and direct analysis of human serum and urine samples. Moreover, the fabrication of NGQDs@VMSF/ITO sensor is convenient, cost-effective, and requires less-complex modification steps, making it a promising candidate for the monitoring of DOX in biological fluids and showing potential application for portable on-site analysis in clinical diagnosis.

## Figures and Tables

**Table 1:** Comparison of the analytical performance of NGQDs@VMSF/ITO electrode with other reported electroanalytical methods for DOX determination.

Sensor Material	Method	Linear Range (μM)	Sensitivity (μA μM*^−^*^1^)	LOD (nM)	Ref.
AgNPs/Chitosan/GCE	SWV	0.103–8.6	0.8613	103	[3]
AuNPs@COFs-MWCNTs/GCE	DPV	0.08–3.0, 3.0–25	1.185	16	[50]
NiCo-BTC MOFs/N-GONRs/GCE	SWV	0.01–1.0, 1.0–80	95.024	6	[51]
MB@MWCNTs/UiO-66-NH_2_/GCE	DPV	0.1–75	0.01829	51	[52]
VMSF/ErGO/GCE	DPV	0.001–20	7.815	0.77	[12]
AuNRDs/1T-MoS_2_/SPE	DPV	0.01–9.50	0.8952	2.5	[1]
NGQDs@VMSF/ITO	LSV	0.005–0.1, 0.1–1	30.4	0.5	This work

AgNPs: silver nanoparticles; GCE: glassy carbon electrode; SWV: square wave voltammetry; DPV: differential pulse voltammetry; AuNPs: gold nanoparticles; MWCNTs: multi-walled carbon nanotubes; NiCo-BTC MOFs: Ni/Co bimetallic metal−organic frameworks; N-GONRs: nitrogen-doped graphene oxide nanoribbons; MB: methylene blue; ErGO: electrochemically reduced graphene oxide; AuNRDs: homogenous and uniform single-crystal gold nanorods; 1T-MoS_2_: metastable 1T phase molybdenum disulfide; SPE: screen-printed electrode.

**Table 2:** Determination of DOX in serum and urine samples.

Sample ^a^	Added (μM)	Found (μM)	RSD (%)	Recovery (%)
Serum	0.100	0.101	0.1	99.0
	0.500	0.499	1.1	100
	1.00	0.990	2.6	99.9
Urine	0.100	0.109	2.4	109
	0.500	0.514	3.8	103
	1.00	0.970	4.3	97.0

^a^ Samples with the addition of known concentrations of DOX were diluted 50 times using PBS (0.01 M, pH = 7). The concentration of DOX was the concentration after dilution.

## Data Availability

The data presented in this study are available on request from the corresponding author.

## Supplementary Materials

The following supporting information can be downloaded at: https://www.mdpi.com/article/10.3390/molecules28186443/s1, Figure S1: SEM and EDS characterizations of NGQDs@VMSF/ITO electrode.
