# Supplementary material for: N-Doped Graphene Quantum Dots Confined within Silica Nanochannels for Enhanced Electrochemical Detection of Doxorubicin
Source: Molecules. 2023 Sep 5;28(18):6443. doi: 10.3390/molecules28186443 (PMC10536127; doi:10.3390/molecules28186443)
Supplement: Supplementary file 1 [file molecules-28-06443-s001.zip › molecules-2566872-supplementary.pdf]

Supporting Information to

N-Doped Graphene Quantum Dots Confined within Silica Nanochannels for Enhanced Electrochemical Detection of Doxorubicin

Chaoyan Zhang [1,†], Xiaoyu Zhou [1,†], Fei Yan [1,*] and Jing Lin [2,*]

[1] Key Laboratory of Surface & Interface Science of Polymer Materials of Zhejiang Province, Department of Chemistry, Zhejiang Sci-Tech University, Hangzhou 310018, China; 202130107346@mails.zstu.edu.cn (C.Z.); 202120104191@mails.zstu.edu.cn (X.Z.)
[2] The First Affiliated Hospital of Guangxi University of Chinese Medicine, Nanning 530200, China
* Correspondences: yanfei@zstu.edu.cn (F.Y.); linj2011@gxtcmu.edu.cn (J.L.)
† These authors contributed equally to this work.

Table of Contents

S1. Materials and Methods

S2. SEM and EDS characterizations of NGQDs@VMSF/ITO electrode

S1. Materials and Methods

S1.1. Chemicals and Materials

ITO coated glass (<17Ω/square, thickness: 100 ± 20 nm) received from Zhuhai Kaivo Optoelectronic Technology (China) was first cleaned by immersing it into 1 M NaOH aqueous solution overnight, and then sonicating in acetone, ethanol, and deionized water for 30 min, respectively. Acetone, ethanol, doxorubicin (DOX), tetraethoxysilane (TEOS), cetyltrimethylammonium bromide (CTAB), potassium ferricyanide ($K_3[Fe(CN)_6]$), 1-aminopyrene, sodium phosphate monobasic dihydrate (NaH_2PO_4), glucose (Glu), ascorbic acid (AA), uric acid (UA), dopamine (DA), amylum, bovine serum albumin (BSA), potassium phthalate monobasic (KHP), and glutathione (GSH) were bought from Aladdin (China). Sodium nitrate ($NaNO_3$), sodium chloride (NaCl), potassium chloride (KCl), and ammonia water were purchased from the Hangzhou Gaojing Fine Chemical Reagent (China). Sodium hydroxide (NaOH), sodium phosphate dibasic dodecahydrate (Na_2HPO_4), Hexaammineruthenium (III) chloride ($Ru(NH_3)_6Cl_3$), nisin, fish sperm DNA were obtained from Macklin (China). Hydrochloric acid (HCl) was bought from Hangzhou Shuanglin Chemical Reagent Co., Ltd. (China). Phosphate-buffered saline (PBS, 0.01 M, pH 7.4) was prepared by mixing NaH_2PO_4 and Na_2HPO_4 aqueous solution in a specific ratio. Human blood serum (healthy man) for real sample analysis was provided by Hangzhou Institute of Occupational Diseases (Hangzhou, China). Human urine samples are obtained from the healthy volunteers. All chemicals were of analytical grade and used as received. And ultrapure water with a resistivity of 18.2 MΩ cm was used throughout the study.

S1.2. Measurements and Instrumentations

Transmission electron microscopy (TEM) investigation was performed on a JEM-2100 microscope (JEOL Ltd., Japan) using an acceleration voltage of 200 kV. Scanning electron microscopy (SEM) measurement was carried out on a FEI Quanta 650 microscope (FEI Ltd., America) using an acceleration voltage of 3 kV. X-Ray photoelectron spectroscopy (XPS) analysis were obtained from a PHI5300 electron spectrometer (PE Ltd, USA) with Mg Kα radiation (250 W, 14 kV). Cyclic voltammetry (CV) and Line sweep voltammetry (LSV) were carried out on a CHI660 electrochemical workstation (PGSTAT302N, Metrohm, Switzerland). A three electrodes system was adopted with bare or modified ITO as the working electrode, an Ag/AgCl (saturated KCl solution) electrode as the reference electrode, and a Pt-sheet electrode (1 cm × 1 cm) as the counter electrode.

S1.3. Synthesis of NGQDs

NGQDs was synthesized using a one-step hydrothermal method according to the previous report [1]. Briefly, 1-aminopyrene (2 mg/mL) was dissolved in 200 mL of ammonia water (0.4 M) and the mixture was heated at 200°C for 2 h. The resulting mixture was then filtered through a 0.22 μm filter membrane to remove large particles. And the filtrate was dialyzed for 24 h using a dialysis bag with a cut-off molecular weight of 500 Da to remove unreacted small substances. After being freeze-dried, NGQDs powder was obtained.

S1.4. Fabrication of VMSF/ITO and NGQDs@VMSF/ITO electrodes

VMSF/ITO was prepared by prehydrolyzing a silica-based precursor solution

consisting of CTAB (1.585 g), TEOS (3.05 mL), $NaNO_3$ (20 mL, 0.1 M, pH = 2.6) and ethanol (20 mL) under stirring for 2.5 h. Bare ITO electrode was immersed in the precursor solution with Ag/AgCl as the reference electrode and a platinum sheet as the counter electrode. A constant current density of −0.7 mA/cm^2 was applied for 10 seconds to obtain the ITO electrode modified with surfactant micelles (SM)-templated VMSF, designated as SM@VMSF/ITO. SM was then extracted using a 0.1 M HCl-ethanol solution to achieve VMSF/ITO.

Confinement of NGQDs into the nanochannels of VMSF was accomplished by convenient electrophoresis according to our previous report. Briefly, VMSF/ITO electrode was immersed into a NGQDs solution (0.3 mg/mL) and underwent for a positive potential of +0.8 V for 10 min, finally obtaining NGQDs@VMSF/ITO.

S1.5. Electrochemical determination of DOX using NGQDs@VMSF/ITO electrode

A series of DOX with known concentrations were added to the 0.01 M PBS solution (pH 7.0) and then NGQDs@VMSF/ITO electrode was immersed into the above detection solution. The enrichment procedure was performed under stirring for 120 s and LSV method was employed to record the electrochemical signals of DOX at room temperature. The cathodic peak currents displayed at −0.6 V were used for the determination of DOX.

S2. SEM and EDS characterizations of NGQDs@VMSF/ITO electrode

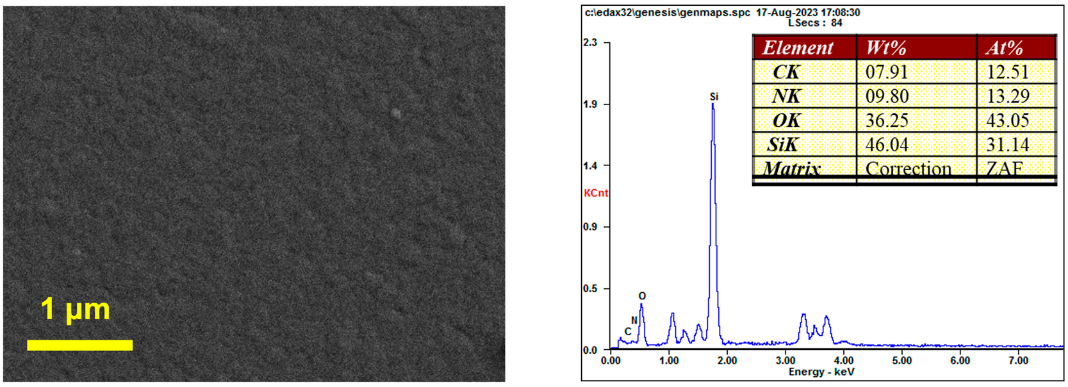

(a) (**b**)

Figure S1. SEM image (a) and energy dispersive spectrometer analysis (b) of NGQDs@VMSF/ITO.

Reference

1. Wan, Y.; Zhao, J.; Deng, X.; Chen, J.; Xi, F.; Wang, X. Colorimetric and fluorescent dual-modality sensing platform based on fluorescent nanozyme. *Front. Chem.* **2021,** *9*, 774486.
